# Comparison of Alternative Meat Inspection Regimes for Pigs From Non-Controlled Housing – Considering the Cost of Error

**DOI:** 10.3389/fvets.2018.00092

**Published:** 2018-06-05

**Authors:** Rikke Koch Hansen, Lisbeth Harm Nielsen, Mahmoud El Tholth, Barbara Haesler, Alessandro Foddai, Lis Alban

**Affiliations:** ^1^Department for Food Safety and Veterinary Issues, Danish Agriculture & Food Council, Copenhagen, Denmark; ^2^Department of Economics, School of Arts and Social Sciences, City University of London, London, United Kingdom; ^3^Department of Hygiene and Preventive Medicine, Faculty of Veterinary Medicine, Kafrelsheikh University, Kafr El Sheikh, Egypt; ^4^Veterinary Epidemiology Economics and Public Health Group, Royal Veterinary College, Hatfield, United Kingdom; ^5^Department of Epidemiological Sciences, Animal and Plant Health Agency, Addlestone, United Kingdom

**Keywords:** surveillance, outdoor production, early warning, *Mycobacterium bovis*, meat inspection, cost of error, pigs

## Abstract

Denmark has not had cases of bovine tuberculosis (bovTB) for more than 30 years but is obliged by trade agreements to undertake traditional meat inspection (TMI) of finisher pigs from non-controlled housing to detect bovTB. TMI is associated with higher probability of detecting bovTB but is also more costly than visual-only inspection (VOI). To identify whether VOI should replace TMI of finisher pigs from non-controlled housing, the cost of error – defined here as probability of overlooking infection and associated economic costs - should be assessed and compared with surveillance costs. First, a scenario tree model was set up to assess the ability of detecting bovTB in an infected herd (*HSe*) calculated for three within-herd prevalences, *WHP* (1, 5 and 10%), for four different surveillance scenarios (TMI and VOI with or without serological test, respectively). *HSe* was calculated for six consecutive 4-week surveillance periods until predicted bovTB detection (considered high-risk periods *HRP*). 1-*HSe* was probability of missing all positives by each *HRP*. Next, probability of spread of infection, *P_spread_,* and number of infected animals moved were calculated for each *HRP*. Costs caused by overlooking bovTB were calculated taking into account *P_spread_*, 1-*HSe*, eradication costs, and trade impact. Finally, the average annual costs were calculated by adding surveillance costs and assuming one incursion of bovTB in either 1, 10 or 30 years. Input parameters were based on slaughterhouse statistics, literature and expert opinion. Herd sensitivity increased by high-risk period and within-herd prevalence. Assuming *WHP*=5%, *HSe* reached median 90% by 2nd* HRP* for TMI, whereas for VOI this would happen after 6th* HRP*. Serology had limited impact on *HSe*. The higher the probability of infection, the higher the probability of detection and spread. TMI resulted in lowest average annual costs, if one incursion of bovTB was expected every year. However, when assuming one introduction in 10 or 30 years, VOI resulted in lowest average costs. It may be more cost-effective to focus on imported high-risk animals coming into contact with Danish livestock, instead of using TMI as surveillance on all pigs from non-controlled housing.

## Introduction

Denmark has been officially free from bovine tuberculosis (OTF) since the first declaration in 1980. The last detected, positive cases of bovine tuberculosis (bovTB) were seen in farmed deer in 1994 (1) and traced back to imported deer. The year of the last case of bovTB in Danish pigs is unknown, but it has not been detected for decades (Personal communication, S. Mellergård, Danish Veterinary and Food Administration, 2017); before that occasional cases occurred (2). BovTB may be found in pigs, in particular if raised outdoors in areas known for presence of bovTB (3). In the United Kingdom, which is not OTF, pigs are found positive for bovTB sporadically; from 2000 to 2010, between 0 and 29 pigs were found positive for bovTB annually (4). This low number is probably a result of a spill-over from infected cattle or wildlife. The main infection pathway for *Mycobacterium bovis* to pigs is by ingestion of infected tissue or contaminated dairy products (5–7), primarily leading to the alimentary form of the disease implying lesions in the lymph nodes in the gastro-intestinal tract including the mandibular lymph nodes. Contrary to cattle, pigs are not believed to excrete *M. bovis* in milk or urine although it cannot be fully ruled out (5, 6, 8). Hence, pigs usually serve as dead-end-hosts for *M. bovis.*

Meat inspection is an essential element of eradication and proving freedom from bovTB (9). In the European Union (EU), traditional meat inspection (TMI) involving palpation and incisions into the mandibular lymph nodes as well as palpation of selected gastro-intestinal lymph nodes have been used to detect bovTB in pigs and cattle during *post mortem* meat inspection for decades. EU Regulation 854/2004/EC introduced the option of visual-only inspection (VOI) of meat for controlled housing pigs, if a risk assessment could prove no or only a negligible increase in risk for human or animal health associated with a change in inspection (10).

Controlled housing refers to indoor production facilities with a high level of biosecurity. EU legislation stipulates a range of requirements for a holding to be considered a controlled housing compartment (11). Holdings not complying with these requirements are considered as non-controlled (i.e., low biosecurity). In Denmark, auditing of the biosecurity status is undertaken by a third party independent auditor at 3 year intervals (12).

In Denmark, two national risk assessments were carried out focusing among others on the effect of omitting palpation and incisions into the mandibular lymph nodes and the palpation of the jejuni lymph nodes (13, 14). Based upon these two assessments, a partial VOI of pigs from controlled housing was implemented implying no routine opening of the heart, no routine incisions into the mandibular lymph nodes, and no routine palpations of the intestinal lymph nodes. In 2013, a third Danish risk assessment evaluated the risk associated with not palpating the lungs (15). In 2016, a full VOI was implemented for indoor-raised finishers after acceptance of equivalence was obtained from important trade partners.

Following a report by the European Food Safety Authority (EFSA) recommending to use VOI of swine to limit the spread of foodborne hazards such as *Salmonella* spp. (16), the EU meat inspection legislation was changed in 2014. Regulation 218/2014/EC stipulates that *post mortem* meat inspection in all swine should be carried out as VOI as the general approach. Hereby, VOI was supposed to replace TMI of all pigs, independent of age or rearing system, unless geographical information, food chain information or findings during *ante mortem* inspection revealed a risk to human or animal health (17). However, the EU Regulation did not foresee that legislation in countries outside the EU may not allow VOI, which is known to have a lower sensitivity of detection of bovTB compared to TMI (16, 18). This has led to negotiations between the EU and the authorities in the USA, during which it has been mentioned that “if an EU Member State requests visual-only *post-mortem* inspection in hogs other than market hogs that have been raised indoors, they must provide supporting documentation and data that supports visual-only inspection in these situations” (19). In fact, bilateral trade agreements based on the requirements set by the EU Regulation from 2004 do not consider VOI for pigs reared under non-controlled housing conditions, because VOI was not allowed in that animal group in the EU before 2014. Therefore, any change to meat inspection of pigs from non-controlled housing must be renegotiated with such trade partners.

The median annual probability of introduction of bovTB to Danish cattle stemming from cattle import and immigrant workers was previously estimated at 0.7%; and import of cattle constituted the far majority of the relatively low probability of introduction (20). Introduction by wildlife was not included in the estimation, as bovTB has never been detected in Danish wildlife (1). The annual number of imports of pigs to Denmark is limited; between 2014 and 2016 imports targeted two to five herds and involved 366 pigs on average (1). Because bovTB should not spread between pigs, the introduction to Danish pigs is unlikely to occur via imported pigs. Introduction of bovTB into non-controlled pig productions through other species than pigs cannot be excluded; and potential transmission pathways include introduction of infected wildlife or other kinds of imported animals.

Denmark consists mainly of islands and there is only one terrestrial border with Germany, which is 68 km long. Germany is considered OTF, however, bovTB cases were found from 2005 to 2008 in cattle and wildlife. Germany is considered free from bovTB in wildlife since November 2009 and in domestic livestock since April 2015 (21). Only few free-range wild boars remain in Denmark; they are mainly found in forest areas northeast of the German border due to migration over the border (22, 23). Due to a concern for the spreading of African swine fever, the Danish authorities have launched an eradication campaign for wild boar not kept under fence in Denmark (http://mfvm.dk/nyheder/nyhed/nyhed/minister-gaar-til-kamp-for-at-hindre-afrikansk-svinepest/). In conclusion, the probability of introduction of bovTB due to immigrating wildlife seems to be very low.

Guard alpacas or llamas are widely used for livestock protection in countries like Australia, because of these animals’ protective behaviour including protection against canids (24). However, these animals may carry bovTB. Between one and 15 camelid premises were identified annually as new incidents of culture-confirmed *M. bovis* in Great Britain in the years from 2004 to 2015 (4). In Belgium, bovTB was recently introduced with imported llamas (Personal communication, S. Welby, Coda-Cerva, 2017). Consequently, the present work takes into account this potential pathway and uses a hypothetical situation, where bovTB is introduced by imported, infected camelids from anywhere in the world.

So far, Denmark has retained TMI for pigs from non-controlled housing due to export requirements. To determine whether VOI could replace TMI of pigs from non-controlled housing, it is necessary to compare the costs of surveillance with the expected cost of error, defined as the probability of overlooking infection, which will then lead to an outbreak, multiplied with the economic consequences associated with an outbreak (25).

The aim of this work was to compare the costs of bovTB surveillance (in the form of meat inspection) with the expected cost of error - associated with each of four ways of undertaking meat inspection in finisher pigs, raised in non-controlled housing systems in Denmark.

## Materials and Methods

### General Overview and Scenarios Studied

Four surveillance scenarios for finishing pigs from non-controlled holdings were investigated in this study:

- Traditional inspection (TMI), which is the current procedure for this group of pigs in Denmark, i.e., the baseline scenario,
- Visual-only inspection (VOI),
- Traditional inspection (TMI) in combination with serological testing of individual pigs,
- Visual-only inspection (VOI) in combination with serological testing of individual pigs.

Scenarios C and D were inspired by the surveillance for *Toxoplasma gondii* and *M. avium* used by one large abattoir company in the Netherlands (26). The serological test assumed in this scenario aims to detect antibodies for *M. bovis*.

**Figure 1:**
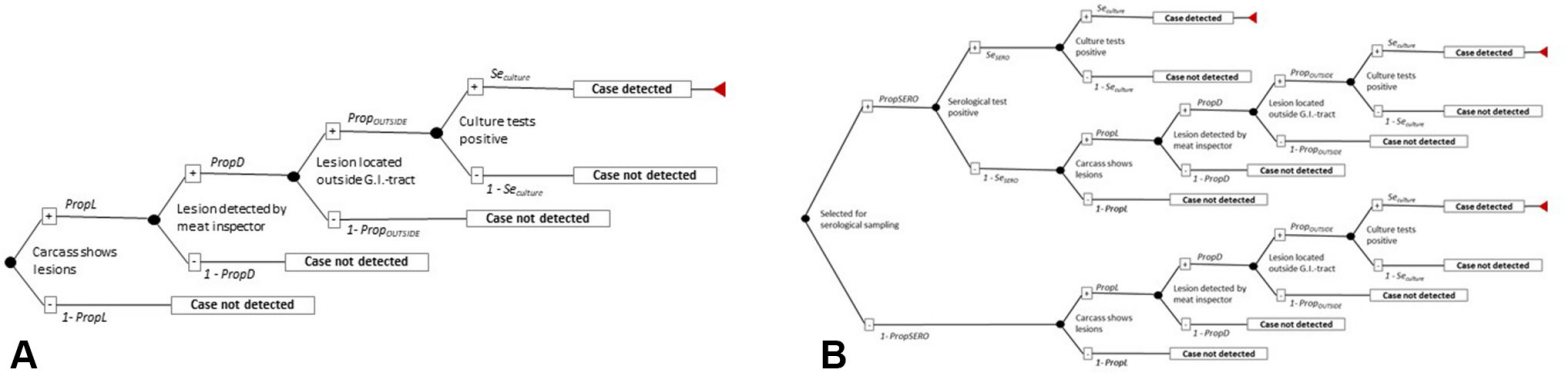
Scenario tree to determine the surveillance unit sensitivity (*SeU* in Eq. 1 and Eq. 2) where **(A)** is applicable for scenario A with traditional meat inspection (TMI) and scenario B with visual-only inspection (VOI), and **(B)** is applicable for Scenario C and Scenario D where the two surveillance methods are supplemented by serological tests. SE = sensitivity, *P* = probability, Prop = proportion.

Each surveillance scenario was investigated by using a stochastic simulation model (scenario tree) which reflected the series of steps required to achieve detection of at least one infected pig from an infected herd (27). The four scenarios are described in brief in Table 1 and depicted in detail in Figure 1.

**Table 1:** Number of tests to take and cost of meat inspection for each of the four scenarios studied of Danish finisher pigs raised under non-controlled housing conditions.

	Price* per test (€)			No. of tests to take			Annual costs(million €)
Scenario	Visual-only Inspection	Traditional Meat Inspection	Serology	Visual-only Inspection	Traditional Meat Inspection	Serology	
A		1.73	-	0	900,000	0	1.560
B	1.2	-	-	900,000	0	0	1.080
C	-	1.73	13.33	0	900,000	4,500	1.721
D	1.2	-	13.33	900,000	0	4,500	1.140

*Based upon information from the two largest abattoir companies in Denmark (Personal communication, L. Bjertrup, Danish Crown, 2016; personal communication, H. B. Lauritsen Tican, 2017).

The calculations of probabilities of overlooking any possible bovTB infected pig [P(T-|D+)] from the first infected herd using different meat inspection regimes were based on inputs fed into the models originating from slaughterhouse statistics, scientific or grey literature, and complementing expert opinion (Table 2). Next, the probability of spread of infection to other herd(s) was estimated (*P_spread_*). The latter probability was multiplied by the estimated costs of consequences in case of spreading (impact of error). Inputs for calculating these probabilities were based on expert opinion gathered by three questionnaires addressed to: (i) Danish pig farmers, (ii) experts of the national pig industry/market, and (iii) European experts on bovTB. The questionnaires can be obtained by contacting the corresponding author.

**Table 2:** Input parameters for calculation of cost of error related to different meat inspection regimes in case of hypothetical outbreak of bovine tuberculosis in Danish pigs, 2017.

**Input parameter**	**Abbreviation**	**Input value / Distribution**	**Source**
Within-herd prevalence	WHP_low_	0.01	(18, 21, 28)
	WHP_medium_	0.05	(3)
	WHP_high_	0.10	(29)
Prop. of positive pigs with lesions	PropL	Beta (14,13)	(9)
Prop. of pigs with lesions located outside digestive tract	Prop_OUTSIDE_	Beta (23, 34)	(3)
Prop. of positive pigs with lesions that are detected	PropD_TMI_PropD_VOI_	Pert (0.47; 0.71; 0.82)Pert (0.142; 0.237; 0.34)	(18, 28, 30)
Se of culture test	Se_CULTURE_	Pert (0.92; 0.95; 0.98)	(18, 28, 30, 31)
Prop. of pigs selected for serological sampling	Prop_SERO_	0.005	Assumed one out of 200
Se of serological test	Se_SERO_	Pert (0.66; 0.72; 0.82)	(32)
Annual prob. of pig being moved between two Danish herds	P_movement_	0.28	53,732 pigs moved out of 188,212 produced on 12 non-controlled herds in 2015
No. of herds receiving infected pigs	N_received_	1 = 74%, 2 = 13%, 3 = 4%, 4 = 9%	Original data from (33)
No. of incursions of bovTB into Danish pigs in 30 years	N_incurse_	1, 3, 30	Chosen by the authors
Prob. of primary consequences, when disease has spread	P_primaryS_	1	Estimate: Interview of farmers
Prob. of activated eradication program, when disease has spread	P_eradicationS_	1	Estimate: Interview of international experts
Prob. of reaction from trade partners, when disease has spread	P_tradeS_	0.15	Estimate: Interview of national experts
Prob. of primary consequences, when disease has not spread	P_primaryNS_	1	Estimate: Interview of farmers
Prob. of activated eradication program, when disease has not spread	P_eradicationNS_	0.3	Estimate: Interview of international experts
Prob. of reaction from trade partners, when disease has not spread	P_tradeNS_	0.05	Estimate: Interview of national experts
Cost of primary consequences	C_primary_	€1.230	Calculated: See equation 5
Cost of activated eradication program per infected herd	C_eradication_	€32.812	Estimate/Calculated: See equation 6
Cost of reaction from trade partners	C_trade_	Pert(€32.5M; €65M; €130M)	Calculated: See equation 7. Assumed Japan shuts down for import of pigs and products thereof for 3/6/12 months and the products meanwhile sold at alternative markets with 25% price reduction (34). Based on export value of € 520M in 2016 (35)

Prob. = probability. Prop. = proportion.

The Palisade software @RISK for Excel version 7.5 was used for the calculations. Simulations were run using 10,000 iterations and Latin hypercube sampling. The convergence of the model was checked every 100 iterations, on any combination of mean, SD, and percentile; for the main outputs. The convergence tolerance was specified at 3%, and the confidence level at 95%.

A list of abbreviations of variables used in the modeling is given in Table 2.

### Data Collection

#### Data on the Investigated Pig Population

In Denmark, most pigs are raised under controlled housing conditions: approximately 17.7 million finisher pigs are slaughtered every year (28). Of those, approximately 900,000 originate from non-controlled housing units (Personal communication, P. Fraas, Friland Food, 2016).

According to different national statistics and data from the independent third party auditor, controlling biosecurity on-farm, 344 holdings were identified as non-controlled finisher herds (28). The average production of a non-controlled housing facility would therefore be 2,616 finisher pigs per year corresponding to a 4 week delivery of around 200 finisher pigs. For this study, a full production cycle of finishers (from birth to slaughtering) was assumed to consist of six deliveries, corresponding to 5.5 months.

#### Literature Data

Literature data regarding the epidemiology of bovTB were obtained using Cab Abstract and Google Scholar abstract search. Moreover, the PRISMA checklist was used (http://prisma-statement.org). The literature search was restricted to English language. No limit on year was used; the newest references were used in general, unless an older one was judged as more relevant. The following search terms were used in the Cab Abstract database: “Bovine Tuberculosis”, “*Mycobacterium bovis*”, “*M. bovis*”, “pig*”, “swine”, “disease prevalence”, “freedom from disease”, “surveillance”, “meat inspection”, “introduction”, and all search combinations thereof, with and without the combination “AND pig* OR swine”. To achieve the most relevant literature, the search was restricted to literature containing the search terms in either the title or abstract, applying the “.ti,ab”-function. Search results were excluded or included in an initial screening based on (1) the title and (2) a skim-read of belonging abstract. The literature database Google Scholar was applied differently; we used Google Scholar for searching titles or authors of literature cited in relevant literature, achieved by the Cab Abstract search. Additional information about legislation was accessed through the internet using public databases covering Danish legislation, EU legislation, and OIE guidelines.

#### Elicitation of Expert Opinion

Three different elicitations of expert opinion were conducted, as explained below. Table 2 summarizes the information elicitated.

##### Elicitation of Expert Opinion From Farmers

Information was collected from Danish pig producers with a history of bovTB suspicion in the herd. During winter 2016/17, a total of 40 pig holdings were imposed official supervision for bovTB suspicion, due to TB-like lesions (defined as granulomas in lymph nodes present outside the gastro-intestinal tract) detected at *post mortem* meat inspection. The normal incidence of this finding is between zero and two holdings per month, and an investigation was undertaken to reveal the reason for the observed increase in suspicions. Allocation of raw peat with tubercles of the avian-related type of tuberculosis called *M. avium* was identified as the cause of the outbreak (29). The owners of the 40 pig holdings under suspicion for bovTB were contacted by employees from their abattoirs to introduce them to the project and to obtain their approval to participate. Contact details of the farmers were supplied by the abattoirs. Opinions of 39 farmers were elucidated by telephone interview using a structured questionnaire, which required between 5 and 20 min each. The questionnaire included questions about possible ways of introduction for *M. bovis*, *M. avium* or the human-related type of tuberculosis *M. tuberculosis* to their farms. The farmers were also asked if they had experienced any economic losses in relation to restrictions, rejected animals or tuberculin test. The questionnaire can be obtained through contact to the corresponding author of the paper.

##### Elicitation of Expert Opinion From National Experts

The second expert opinion elicitation aimed at collecting data on the economic consequences of an introduction of bovTB to the national pig herd. This questionnaire was addressed to 14 national experts with knowledge of trade in live animals and animal products from the Danish Agriculture & Food Council (*n* = 9), the Ministry of Environment and Food of Denmark (*n* = 3), and animal trade companies (*n* = 2). The 14 experts were interviewed in groups or individually, either face to face or by phone using a pre-developed questionnaire. Each interview lasted from 30 to 60 min, and all interviews were carried out by the same two interviewers. The questionnaire can be obtained through contact to the corresponding author.

##### Elicitation of Expert Opinion From European Experts

The third elicitation was addressed to European experts with knowledge about bovTB and its potential economic consequences. Experts were chosen among EU Member States that had experienced cases of bovTB during the last 10 years; a key criterion for expert selection was in-depth knowledge of recent or current outbreak(s). An invitation e-mail, with the interview questionnaire attached, was sent to potential experts from Austria, Belgium, Germany, Italy, Portugal, United Kingdom, and France. Seven experts representing Belgium (*n* = 1), United Kingdom (*n* = 1), Austria (*n* = 4) and Portugal (*n* = 1) agreed to participate and were  linterviewed by Skype for Business or by phone. The answers from each expert were recorded. Thereafter, the completed questionnaire with the individual answers was sent to the participants, to give them a chance to adjust their answers. The questionnaire can be obtained through contact to the corresponding author.

### Scenario Trees and Surveillance Unit Sensitivity (*SeU*)

A scenario tree model was used to simulate each surveillance scenario (A, B, C, and D) and its respective probability of detecting a bovTB infected pig from the first infected herd or holding (the surveillance unit sensitivity or *SeU*) using the input parameters summarised in Table 2.

For instance, for surveillance scenario A and B, the *SeU* represented the combined probabilities that an infected finisher pig arriving at the abattoir from the first index herd, was correctly classified as positive by the meat inspection used (TMI versus VOI). Then according to Figure 1A and Equation 1, the *SeU* of surveillance scenarios A and B, was calculated as:

SeU = PropL * PropD * Prop_OUTSIDE_ * Se_CULTURE_ (1)

*PropL* was the probability that lesions were present in an infected animal at the time of slaughter. Since no information was available for pigs, this probability was based upon an Australian study in cattle, in which lesions were present in 13 out of 25 bovTB-infected bovine tuberculin-positive animals subjected to detailed necropsy (9). Hence, a Beta [13 + 1, 25–13 + 1] distribution was used.

*PropD* was the probability that the meat inspector detected lesions on an infected pig by TMI or VOI. No information was available for pigs and therefore information from cattle was used. Hence, these probabilities were set as Pert distributions according to EFSA (18, 30) and Calvo-Artavia et al. (31). Hence, for TMI the *PropD* was set to Pert(0.47;0.71;0.82) and for VOI: Pert(0.14; 0.24; 0.34).

In Denmark, only lesions located outside the gastro-intestinal tract are considered suspects of bovTB by the authorities (32). In response, samples are only submitted to culture for bovTB, if the lesions are located outside the gastro-intestinal tract (Personal communication J.S. Oberthon, Danish Veterinary and Food Administration, 2017). Therefore, an extra node (Figure 1) called *Prop_OUTSIDE_* was inserted into equation 1, representing the probability that the lesion was located outside the gastro-intestinal tract and consequently was submitted for culture test. Information about *Prop_OUTSIDE_* originated from Bailey et al. (3), who found that among 55 bovTB infected pigs (for which information was available about location of the lesion), 16 (29%) had lesions in the thoracic cavity and six had generalized lesions (11%). Thus, the proportion of pigs with lesions, located outside of the gastrointestinal tract was 40% and was simulated as: *Prop_OUTSIDE_ =* Beta [(16 + 6) +1, 55 – (16 + 6) +1].

*Se_CULTURE_* was the sensitivity of the culture test and was set to Pert (0.92;0.95;0.98) according to (18, 30, 31).

For surveillance scenarios C and D, *SeU* was calculated according to Figure 1B and Equation 2 to take into account that in these scenarios an infected pig could be found positive for bovTB in three different ways: (i) because it was tested by serology and resulted positive (in serial order) to both antibodies and culture, or (ii) because it resulted negative for antibodies, but could be still detected by lesions (present outside of the gastro-intestinal tract) and culture, or (iii) because of lesion(s) present outside the gastro-intestinal tract, which lead to submission of a sample for culture.

*SeU =* [(Prop_SERO_ * Se_SERO_* Se_CULTURE_)* +* (Prop_SERO_ * (*1 - Se_SERO_*) * PropL* PropD * Prop_OUTSIDE_ * Se_CULTURE_) *+* (*1-PropSERO*) * PropL * PropD * Prop_OUTSIDE_ * Se_CULTURE__)_)] (2)

Where, *Prop_SERO_* was the probability that the processed infected pig was tested in the serological test, and this was set to 0.005, reflecting the assumption that only one out of 200 pigs delivered in a batch would be tested, irrespective of whether TB-like lesions were present or not. 1*-Prop_SERO_* was the probability that the infected pig was not tested by serology, set to 0.995.

*Se_SERO_* was the sensitivity of the serological test and was set to Pert(0.66;0.72;0.82) based upon Cardoso-Toset et al. (33). The remaining elements are described in Eq. 1. Moreover, it was assumed that infected pigs (carrying *M. bovis*) had already seroconverted at the day of slaughter.

### Temporal Herd Sensitivity in the First Infected Herd

The temporal herd sensitivty (*HSe*) for the first infected herd was estimated using the hypergeometric approximation (34, 35), which allows taking into account sampling without replacement from a finite population of animals. Thus:

*HSe =* 1- [1- SlauPigs/Nfinishers * SeU] ^(WHP * Nfinishers)^ (3)

Where *Nfinishers* represented the annual average number of finishers produced, per production circle, in the first infected herd (≈ 2,600 finishers/year corresponding to 1,200 finishers in a production cycle of 5.5 months). *SlauPigs* represented the population of slaughtered pigs from the infected herd (from finisher group) by each *HRP*. Therefore, *SlauPigs* increased by 200 processed pigs after each of the six surveillance periods (e.g., by 1st* HRP* = 200 pigs, by 2nd* HRP* = 200 pigs +200 pigs, and so on). This implied that all pigs present at day 1 of disease introduction and raised for slaughtering were considered tested by meat inspection after six *HRPs* (*SlauPigs*/*N_finishers_* = 1, by the 6th* HRP*)

*WHP* was the within-group prevalence and represented the probability that the processed pig was itself infected. *WHP* was set to three different values reflecting a low (0.01), medium (0.05) or high within-herd prevalence (0.1) and one sub-scenario was run for each of these values. It was assumed that the disease could cluster with homogenous prevalence, within different groups of the same infected farm. Thus, within-herd prevalence *WHP* also = within group (finisher or other) prevalence. The *WHP* figures were chosen based upon (3) who found 52 United Kingdom pig premises with a total of 112 bovTB-infected pigs yielding an average of 2.2 infected pigs per premise. Moreover, in half of the infected herds (for which information about herd size was available) there were fewer than 11 sows or 101 pigs, whereas 18% of the premises were large with more than 200 sows or 2,000 pigs (3).

In this way, the temporal *HSe* was calculated (36–38) in the first infected herd after each *HRP*. Hence, the *HSe* increased with the number of pigs processed and with the number of *HRPs* elapsed since disease introduction.

At the same time, this approach allowed to relate the time required for detection (when *HSe* reaches a high value such as >90%) with the probability of disease spread (*P_spread_* see below) which could occur meanwhile, before the first infected herd is put under restrictions (due to detection of at least one infected pig at the abattoir).

### Probability of Spread During the High-Risk Period(s)

Apart from the pigs slaughtered during the different *HRPs*, it was assumed that in the first infected herd also 1,200 growing pigs, gilts and sows (*N_other_*) would be present. It was assumed that “if” the farmer moved pigs at least once by the investigated *HRP(s)*; he/she could move up to 100 pigs of the* N_other_* animals (*n_moved_*) to other herds for breeding for a *HRP*. The probability of spread by each *HRPs*, called *P_spread_*, was calculated from Equation 4:

*P_spread_ =* 1 – [1 – (P_movement_ * n_moved_/N_other_)] ^(WHP *^^Nother^^)^ (4)

*P_spread_* was based on the hypergeometric approximation (34) and represented the probability of moving at least one infected pig to at least another pig herd by the investigated* HRP(s)*.

*P_movement_* was the probability that the infected herd moved pigs to at least one other herd during at least one elapsed *HRP*. Data from movement of individual pigs from 12 Danish non-controlled pig herds collected during 2015 showed an annual probability of pigs being moved of 28%. This corresponds to 2.5% average probability per *HRP* of 4 weeks. Thus,* P_movement_* = 1 – (1–0.025) ^number of elapsed *HRPs*^

Therefore, *HSe* will increase with the number of *HRPs* elapsed since disease introduction, whereas *P_spread_* depends (also) upon whether and when animals are moved out of the herd and into other premises. For instance, after three *HRPs* at least one movement could occur with probability = *P_movement_*, and the farmer could move 100 animals in one, two or three *HRPs.* Accordingly, if movement(s) occurred, the number of moved animals (*n_moved_*) by three *HRPs* was set with a uniform distribution between 100 (minimum number of animals moved only in one *HRP*) and 300 (maximum number of moved animals in all the three *HRPs*). Thus, *Pmovement* and *n_moved_/N_other;_* represented probabilities of two conditional events respectively: (a) the farmer moving at least once during the investigated *HRP* periods, and (b) the probability that an infected animal was moved to another herd. The latter was repeated for each of the infected animals (WHP * N_other_) present in the *N_other_* group.

Additionally, it was assumed that the infected animals introduced to the secondary infected herd were not further moved, and thus, the *P_spread_* was calculated only for the first infected index herd. This is a reasonable assumption, since the moved infected pigs should not cause infection in the herd mates in the receiving herd.

Moreover, once the first infected herd was detected, it was assumed that all secondary infected herds would be traced and eradication could be achieved; in the first infected herd as well as the secondary infected herds. To describe the number of secondary infected herds, *N_received_*, the data from an outbreak of avian TB occurring in Danish pigs in the winter of 2016–17 was used (29).

The number of infected pigs moved by the elapsed *HRP(s)*, if movement took place, was calculated as Poisson(*n_moved_* * *WHP*).

### Economic Impact

The economic impact stemming from an outbreak of bovTB was estimated considering the economic costs carried by the pig producers and the abattoirs. These costs related to handling of the primary infected herd, eradicating infection from all infected herds, and impacts on trade. All cost estimates were in Euros. *C_primary_* included costs related to a primary infected herd under suspicion due to (1) carcasses rejected at the abattoir, *C_rejected_*, (2) tuberculin testing of pigs in suspicious herds, *C_tuberculin_*, (3) extra costs to meat inspection due to TMI replacing VOI, *C_ΔMI_*, and (4) compensation to farmers when the day of slaughter is moved forward, *C_compensation_*, due to unsolved suspicion of bovTB (Equation 5). Although the compensation covered the lost income of the farmers, this cost was added to account for inadequate use of the production facilities and hereby constituted a cost to the abattoir.

(5) $$C_{primary}=\left(C_{rejected}+C_{tuberculin}+C_{\Delta MI}+C_{compensation}\right)$$

Costs of eradication (defined as testing, culling and trace-back of infection in a suspect herd) of an outbreak, *C_eradication_* were based upon the costs experienced per positive herd in the interviewed countries and a comparison of their eradication programs and the eradication program described in the Danish legislation. Belgium had a much more comprehensive eradication program than Denmark. The Austrian program was also considered wider-ranging, while the eradication program in United Kingdom matched the descriptions in the Danish legislation. Costs per infected herd were therefore assumed as a weighted average of the costs in Belgium, *C_eradication BE_*, Austria, *C_eradication AT_*, and United Kingdom, *C_eradication UK_*, and where the weights were chosen by the authors based upon the information provided by the experts (Equation 6).

(6) $$C_{eradication}=\frac{0.5*C_{eradication\;BE}+0.75*C_{eradication\;AT}+C_{eradication\;UK}}{3}$$

The trade impact related to reactions from trade partners, *C_trade_*, included the total loss of income, *Loss*, in case of market shutdown, where the product would subsequently be sold at alternative, less attractive markets outside Denmark, during the time of the shutdown, *T_shutdown_*. The interviews with experts revealed that Japan was viewed as an important, sensitive market. Experts could not say precisely *if* or *how* Japan would react to findings of bovTB in Danish pigs, but a market shutdown could not be excluded. It was impossible to say which products would be influenced, the length of the shutdown and what would trigger a reaction. In a mild case, only export of live pigs was expected to be influenced. In a worst case, export of live pigs, pork products, live cattle, beef and dairy productions could potentially be influenced. Further, other countries, such as China, South Korea and Russia (the latter market under the assumption of being reopened in the future) could potentially deny imports because of bovTB. To meet these uncertainties, it was decided to describe the potential economic consequences of a market reaction as a Pert distribution, based on a situation, where Japan closed its market for import of live pigs, pork products and by-products from pigs, for 3 (minimum), 6 (mode) or 12 months (max) combined with a 25% price reduction resulting from having to sell products on a less attractive market as suggested by other authors (39) (Equation 7). Costs related to loss in income in case of a national consumer reaction were also considered. However, this was left out based on inputs from the experts consulted.

(7) $$C_{trade}=Loss*T_{shutdown}$$

The total costs, *Costs*, depended on the probability of further spread of bovTB (*P_spread_*) during the *HRP* elapsed from introduction to detection as well as the three different costs elements: cost of (i) infection in a herd (*C_primary_*), (ii) eradication due to the number of secondary infected herds (*C_eradication_*) and (iii) trade restrictions (*C_trade_*) with their respective probabilities, *P*, depending on spread (*S)*, or no spread (*NS)*.

*Costs ={ P*_*spread*_ * [(P_*primaryS*_ * C_*primary*_*) + ( P*_*eradicsationS*_ * C_*eradication*_*) + (P*_*TradeS*_ * C_*Trade*_*)]} + {(1-P*_*spread*_) * [(P_*primaryNS*_ * C_*primary*_*)+ (P*_*eradicationNS*_ * C_*eradication*_* ) + (P*_*TradeNS*_ * C_*Trade*_*)]}* (8)

Please see Table 2 for a description of the individual components of the formula. *P_primaryS_* and *P_primaryNS_* were set to 1, reflecting that there would always be costs for the primary cases, irrespective of whether there would be spreading or not to other herds. Moreover, *P_eradicationS_* was set to 1, reflecting that an eradication would always be activated, if infection had spread.

Next, the cost of error, *CostError*, was calculated as the probability of having overlooked all cases of bovTB, (*1-HSe*), by the elapsed HRP(s) , multiplied by the *Costs,* estimated in equation 8:

CostError = (1-Hse)*Costs (9)

Finally, the surveillance costs, *CostSurv,* for each scenario were calculated considering the costs related to meat inspection and serology described in Table 1. A time frame of 30 years was chosen. *CostSurv* were added to *CostError,* while taking into account that *CostSurv* would incur every year, whereas *CostError* would only occur, if an incursion of bovTB into Danish pigs took place. Next, the average costs per year, *CostAverage_years_,* were calculated (Equation 10). Here, three different assumptions regarding frequency of incursion, *N_incurse_*, of bovTB in 30 years were used: 30 (corresponding to one disease incursion every year), three (every 10 years) and one (every 30 years). These numbers were chosen to reflect that more than 30 years have passed since the last incursion of bovTB into Denmark (1). This enabled an identification of the most cost-effective of the three scenarios in the long term.

*CostAverage_years_*= (CostSurv * 30 + CostError * *N_incurse_*)/30 (10)

## Results

### Probability of Detection and Spread of bovTB

**Figure 2:**
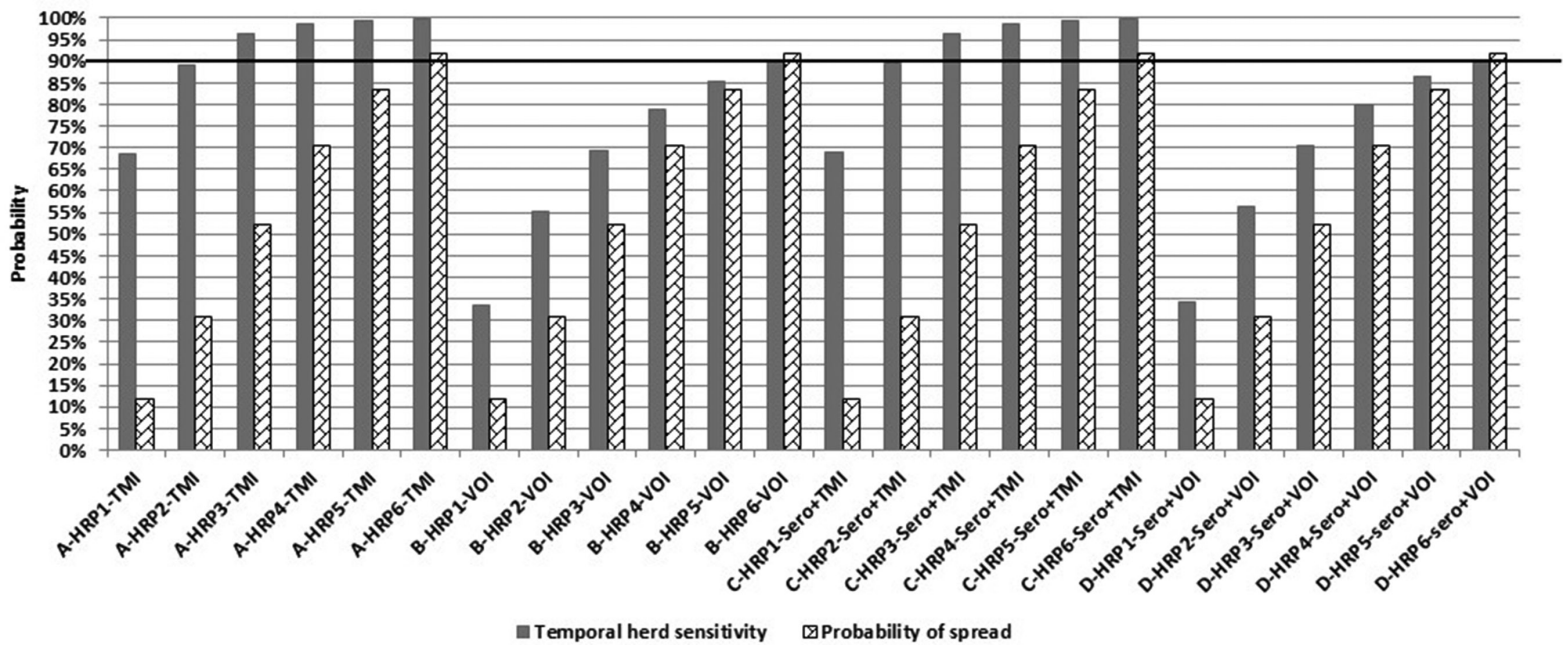
Display of the temporal herd sensitivity (*HSe*) for detection of bovine tuberculosis at meat inspection of finisher pigs delivered from the first infected herd, divided according to number of elapsed surveillance periods (*HRP*), and according to four different kinds of meat inspection. The associated probability of spread of bovine tuberculosis (*PSpread*) from the first infected pig herd to one or more other pigs herds, is also reported assuming that the pig producer could move pigs (between 100 to 600) to other premises, by one or more (up to 6) high risk periods (*HRP/s* of 4 weeks each) while assuming a within-herd (and within-group) prevalence of 5% in the first infected pig herd.

According to the simulations, *HSe* increased by *HRP* and *WHP* (Table 3). Hence, the higher the probability of infection, the higher the probability of detection, due to more “chances” of finding a case of bovTB at meat inspection. Assuming *WHP* = 5%, *HSe* reached 90% by the 2nd* HRP* for TMI, whereas for VOI this would only happen after the 6th* HRP*. Serology had only a limited impact on *HSe* (Figure 2). This is most likely because we assumed that only one animal would be sampled per batch, while at the same time assuming a *WHP* of 5%. For other infections, resulting in a higher *WHP*, one sample per batch may very well be sufficient to detect positive batches.

**Table 3:** Estimated ability to detect a hypothetical case of bovine tuberculosis in the index pig herd (herd sensitivity, *HSe*) for each of four meat inspection regimes, divided according to within-herd prevalence and time period* (*HRP*) after introduction, Denmark, 2017.

	**Herd sensitivity (%) while assuming a within-herd prevalence of**		
**Scenario A Traditional meat inspection**	**1%**	**5%**	**10%**
4 weeks	21 (13, 29)	69 (51, 83)	89 (77, 97)
8 weeks	38 (25, 51)	89 (76, 97)	98 (95, 100)
12 weeks	51 (35, 66)	96 (89, 100)	100 (99, 100)
16 weeks	62 (44, 77)	99 (95, 100)	100 (100, 100)
20 weeks	70 (52, 85)	99 (98, 100)	100 (100, 100)
24 weeks	76 (59, 90)	100 (99, 100)	100 (100, 100)
**Scenario B Visual-only inspection**			
4 weeks	8 (5, 12)	33 (21, 47)	55 (38, 72)
8 weeks	15 (9, 22)	55 (38, 72)	79 (61, 92)
12 weeks	22 (13, 32)	70 (51, 85)	90 (76, 98)
16 weeks	28 (17, 40)	79 (61, 92)	95 (85, 99)
20 weeks	34 (21, 48)	85 (70, 96)	97 (91, 100)
24 weeks	39 (25, 54)	90 (76, 98)	99 (94, 100)
**Scenario C Serology and traditional meat inspection**			
4 weeks	21 (14, 30)	69 (53, 83)	89 (77, 97)
8 weeks	38 (26, 51)	90 (77, 97)	99 (95, 100)
12 weeks	52 (36, 67)	96 (89, 100)	100 (99, 100)
16 weeks	62 (45, 78)	99 (95, 100)	100 (100, 100)
20 weeks	70 (53, 85)	99 (98, 100)	100 (100, 100)
24 weeks	77 (59, 90)	100 (99, 100)	100 (100, 100)
**Scenario D Serology and visual-only inspection**			
4 weeks	8 (5, 12)	34 (22, 48)	56 (39, 72)
8 weeks	16 (10, 23)	56 (39, 73)	80 (63, 93)
12 weeks	23 (14, 33)	71 (53, 86)	90 (78, 98)
16 weeks	29 (18, 41)	80 (63, 93)	95 (87, 100)
20 weeks	35 (22, 49)	86 (72, 96)	98 (92, 100)
24 weeks	40 (28, 55)	90 (77, 98)	99 (95, 100)

*Finisher pigs are delivered to slaughter every 4 weeks.

The probability of spread of infection increased by *HRP* – due to moving animals - and by the assumed *WHP* (Table 4). The median number of moved infected animals was low (median values 1–3), when a *WHP* of 1% was assumed, and higher (median values 10–35) when a* WHP* of 10% was assumed (Table 4). If assuming a *WHP* of 5%, the median *P_spread_* was 27% after the 2nd* HRP* involving a median of seven infected animals, and likewise after the 6th *HRP*, the median *P_spread_* was 89% and involved a median of 17 infected animals (Table 4).

**Table 4:** Median probability of spread^a^ of bovine tuberculosis (bovTB) from one hypothetical infected pig herd to one or more pig herds as well as number of moved infected pigs, divided according to time period (*HRP*) after introduction, while assuming a within-herd prevalence of 1, 5 or 10%

	**Median probability (5th; 95th percentiles) of spread of bovine tuberculosis and number of infected pigs moved to other premises**					
	**Within-herd prevalence 1%**		**Within-herd prevalence 5%**		**Within-herd prevalence 10%**	
**High-risk period**	**Probability of spread **(in %)	**No. of moved infected animals**	**Probability of spread (in %)**	**No. of moved infected animals**	**Probability of spread (in %)**	**No. of moved infected animals**
4 weeks	2.5 (2.5, 2.5)	1 (0, 3)	11.8 (11.8, 11.8)	5 (2, 9)	22.1 (22.1, 22.1)	10 (5, 15)
8 weeks	7.2 (5.1, 9.2)	1 (0, 4)	31.0 (22.9, 38.3)	7 (3, 13)	52.4 (40.5, 62.0)	15 (8, 23)
12 weeks	13.7 (7.8, 19.3)	2 (0, 5)	52.1 (33.2, 65.7)	10 (4, 18)	77.0 (55.4, 88.2)	20 (9, 33)
16 weeks	21.6 (10.5, 31.4)	2 (0, 6)	70.4 (42.7, 84.8)	12 (4, 22)	91.2 (67.1, 97.7)	24 (10, 42)
20 weeks	30.4 (13.4, 44.3)	3 (0, 7)	83.6 (51.2, 94.6)	15 (5, 27)	97.3 (76.2, 99.7)	30 (11, 51)
24 weeks	39.6 (16.3, 56.8)	3 (0, 8)	91.9 (58.8, 98.5)	17 (5, 32)	99.4 (83.0, 100.0)	35 (11, 61)

a: Probability that the farmer moves at least one infected animal to at least one secondary Danish pig herd. For example: by the end of the second *HRP* (by 8 weeks) the probability that the farmer moves at least one infected animal to at least one secondary pig herd is 7.2% (5.1%; 9.2%) when assuming a within-group (*N_other_* = 1,200 animals) animal prevalence of 1% in the source index farm. After *HRP* 1 only one estimate for the probability of spread is available, because only 100 animals out of 1,200 in the *N_other_* group were assumed could be moved (no uniform distribution used to simulate *n_moved_/N_Other_* in Eq. 4).

Figure 2 displays the temporal herd sensitivity (*HSe*) for detection of bovine tuberculosis at meat inspection of finisher pigs delivered from the first infected herd, divided according to number of elapsed surveillance periods (*HRP*), and for each of the four scenarios. The associated probability of spread of bovine tuberculosis (*P_spread_*) from the first infected pig herd to one or more other pig herds, is also reported. Here, it was assumed that the pig producer could move between 100 and 600 pigs to other premises, by one or more (up to six) high-risk periods (*HRP/s* of 4 weeks each), while assuming a within-herd (and within-group) prevalence of 5% in the first infected pig herd. It is noted in Figure 2 that for TMI (Scenario A and C) *HSe* was much higher than *P_spread_*, implying that infection would be more likely to be detected in the herd of origin than being spread by the elapsed *HRP(s)*. This was also seen for scenario B and D involving VOI, however, for those two scenarios the difference between *HSe* and *P_spread_* was smaller, implying a higher probability of spread of infection before detection.

### Economic Impact

The annual costs of surveillance, *CostSurv,* in the form of meat inspection amounted to €1.56 million for scenario A, €1.08 million for scenario B, €1.72 million for scenario C, and €1.14 million for scenario D (Table 1).

According to the experts, the primary costs related to a pig farm under suspicion for bovTB, *C_primary_*, amounted to €1,230, followed by €32,812 in eradication costs for one herd, when a case is confirmed. According to the simulations, the eradication costs in case of spreading to one or more other herds amounted to a median value of €82,250 (90% probability interval: €67,285; 150,364). The potential negative impact on trade, *C_trade_*, amounted to a median value of €65 million (90% probability interval: €32.5 to €130.0) (Table 2).

In Table 5, the average annual cost assuming one incursion of bovTB into Danish pigs per 1, 10 or 30 years, *CostAverage_years_*, is shown, while assuming a *WHP* of 5%. The highest costs were seen, when it was assumed that there would be one incursion of bovTB into Danish pigs every year, and here the scenarios A and C - involving TMI with and without use of serology - were associated with the lowest annual costs. However, if it was assumed that there would only be an incursion every 10 or 30 years, then the lowest average annual costs were seen for the scenarios B and D involving VOI. In those cases, the annual costs were close to the annual costs of meat inspection.

**Table 5:** Average annual costs related to surveillance in the form of meat inspection (divided according to four different regimes*) and costs related to an outbreak while assuming one incursion of bovine tuberculosis into the Danish pig population in 1, 10 or 30 years and a subsequent within-herd prevalence 5% in the first infected pig farm.

**High-risk period**	**Average annual cost in million € (90% probability interval)**		
	**1** year	**10** years	**30** years
**Scenario A**			
4 weeks	3.02 (2.40–4.00)	1.71 (1.64–1.80)	1.61 (1.59–1.64)
8 weeks	2.32 (1.89–3.01)	1.64 (1.59–1.71)	1.59 (1.57–1.61)
12 weeks	1.93 (1.67–2.38)	1.60 (1.57–1.64)	1.57 (1.56–1.59)
16 weeks	1.70 (1.59–1.93)	1.57 (1.56–1.60)	1.56 (1.56–1.57)
20 weeks	1.67 (1.60–1.78)	1.56 (1.56–1.58)	1.56 (1.56–1.57)
24 weeks	1.58 (1.56–1.62)	1.56 (1.56–1.57)	1.56 (1.56–1.56)
**Scenario B**			
4 weeks	4.22 (3.02–5.77)	1.39 (1.27–1.55)	1.18 (1.14–1.24)
8 weeks	4.05 (2.84–5.83)	1.38 (1.26–1.56)	1.18 (1.14–1.24)
12 weeks	3.71 (2.52–5.56)	1.34 (1.22–1.53)	1.17 (1.13–1.23)
16 weeks	3.31 (2.21–5.15)	1.30 (1.19–1.49)	1.15 (1.12–1.22)
20 weeks	2.86 (1.89–4.42)	1.26 (1.16–1.41)	1.14 (1.11–1.19)
24 weeks	2.40 (1.62–3.73)	1.21 (1.13–1.35)	1.12 (1.10–1.17)
**Scenario C**			
4 weeks	3.18 (2.55–4.11)	1.87 (1.80–1.96)	1.77 (1.75–1.80)
8 weeks	2.42 (2.04–3.08)	1.79 (1.75–1.86)	1.74 (1.73–1.77)
12 weeks	2.09 (1.83–2.53)	1.76 (1.73–1.80)	1.73 (1.72–1.75)
16 weeks	1.86 (1.75–2.09)	1.74 (1.72–1.76)	1.73 (1.72–1.73)
20 weeks	1.83 (1.76–1.95)	1.73 (1.73–1.74)	1.72 (1.72–1.73)
24 weeks	1.74 (1.72–1.78)	1.72 (1.72–1.73)	1.72 (1.72–1.72)
**Scenario D**			
4 weeks	4.23 (3.05–5.76)	1.45 (1.33–1.60)	1.24 (1.20–1.29)
8 weeks	4.05 (2.87–5.77)	1.43 (1.31–1.60)	1.24 (1.20–1.29)
12 weeks	3.67 (2.54–5.47)	1.39 (1.28–1.57)	1.22 (1.19–1.28)
16 weeks	3.25 (2.17–5.01)	1.35 (1.24–1.53)	1.21 (1.17–1.27)
20 weeks	2.81 (1.90–4.27)	1.31 (1.22–1.45)	1.20 (1.17–1.24)
24 weeks	2.44 (1.68–3.70)	1.27 (1.19–1.40)	1.18 (1.16–1.23)

*Please see Table 3 for a description of the four scenarios representing four different meat inspection regimes.

## Discussion

### Key Finding of Study

The work undertaken illustrates a hypothetical situation, where bovTB is introduced to Danish non-controlled pigs, and where a subsequent detection by different surveillance approaches has economic consequences depending on the spread of disease before detection, control measures implemented thereafter, and reaction of trade partners. These findings are dependent on critical assumptions. First, it was assumed that bovTB is introduced and detected in this category of pigs and not first in other species, such as cattle or deer. If the disease is detected in cattle or wildlife, it is expected to activate an eradication programme and no cost of error related to overlooked cases in pigs should occur. The other assumption is that the disease is detected at some point. The cost of error would only occur, when the disease is detected. Until detection, the cost of overlooking positive cases would be zero, because no clinical symptoms and production costs are expected. With pigs assumed to be acting as dead-end hosts, there is also a probability that a disease incursion would die out and not be detected. In that case, there would be no cost of error from overlooking all positive cases.

Once detected, there may be substantial costs due to a market reaction to bovTB in pigs. The highest cost of error occurred for the two scenarios assuming VOI. However, considering the surveillance costs, the scenarios were more similar. The total economic impact was comparable across the four scenarios, but stemmed from different sources of costs. This made the different options more or less sensitive to measures that increase economic efficiency. To determine the future surveillance strategy for bovTB in pigs, we included the probability of incursion of bovTB into Danish pigs by running three scenarios, assuming one incursion every year as well as every 10 or 30 years, where the two latter time periods reflect the current situation in Denmark, where bovTB has been absent for more than 30 years. The average annual costs was lowest for the scenario involving VOI, which would advocate for a change to VOI on pigs from non-controlled housings, while knowing that there is a possibility for higher cost of error in an individual year, if the disease is introduced.

Denmark has not had any cases of bovTB for more than 30 years, and if infection should enter the country, the prevailing opinion is that it would be via imported cattle. Therefore, VOI could replace TMI in finisher pigs raised under non-controlled housing conditions. This was also the conclusion of EFSA (18). Hence, countries (or regions within countries) without bovTB would be able to replace TMI with VOI, especially because bovTB is not considered meat-borne, and the role of meat inspection is to detect bovTB to maintain animal health, and pigs may be considered dead-end hosts.

### Shortcoming and Limitations of the Modelling Approach

The main shortcoming was related to the limited knowledge of bovTB in swine. This was also found by EFSA (16) that used a qualitative approach, when assessing the value of the cuts into the mandibular and gastro-intestinal lymph nodes of pigs. Similarly, Ellebroek et al. (40) elicited expert opinion to compensate for the lack of published data regarding the behaviour of *M. bovis* and the effect of meat inspection on the probability of detection of *M. bovis* in pigs. To take into account this large uncertainty, we chose to use scenario analysis – whereby the effect of various scenarios could be studied. This approach was expected to show more directly the impact of a given parameter on the outcome compared to using a sensitivity analysis. In the following, the parameter values for *WHP, PropL, PropD, Prop_OUTSIDE_, Se_SERO_*, *P_spread_*, *C_eradication_* and *C_trade_* are discussed with respect to eventual short-comings and limitations.

If *M. bovis* entered a pig herd, intra-species disease transmission would not be expected, and therefore the simple modelling applied can be justified. A high within-herd prevalence (*WHP*) could be expected, if pigs were fed bovTB infected material e.g., in the format of unpasteurized milk or whey. A medium *WHP* could be expected, if the disease was introduced by another species permanently present, e.g., infected wildlife or guard llamas, kept among the pigs for example through the oral-faecal route (41). To account for uncertainty, we used three different *WHPs* for the initial modelling, and used a *WHP* of 5% when illustrating the economic consequences.

The probability of lesions being present *PropL* was described using data from an Australian study on cattle, because no studies have been conducted in swine. This implies that there is uncertainty around this input. According the Australian study, lesions were only present in around half of the bovTB cases (9), which is overall speaking in line with (7) and (40).

The inspector’s ability to detect TB like lesions (*PropD*), known as the sensitivity of the meat inspection (42) may vary among abattoirs and inspectors. In addition, no standard method for valuation of the sensitivity of VOI and TMI has been developed, imposing uncertainty on the applied values for sensitivity (18). The experience in Denmark is that outbreaks of avian tuberculosis in pigs due to use of peat for young pigs can be detected by VOI (Personal communication J.S. Oberthon, Danish Veterinary and Food Administration, 2017). Still, there is agreement that VOI is associated with a lower ability to detect bovTB than TMI (13, 14, 43), but it is unknown how much lower the sensitivity is. Moreover, the meat inspection sensitivity is most likely affected by the line speed, which varies between slaughter plants and countries, as well as the number of infected carcasses and the age of the slaughtered animals. A larger prevalence of TB-like lesions and older animals increase the sensitivity, where younger stock and a lower prevalence are known to decrease the sensitivity and thereby increase the probability of overlooking any positive case (30, 44). However, the apparent higher risk in sows may also be a result of a longer lifespan, whereby the probability of infection is increased. For reasons of simplicity, the effect of age was ignored in the present analysis.

Regarding *Prop_OUTSIDE_*: Whether a positive case was detected and sent for culture testing, at *post mortem* inspection or not, relied on several factors. First, the lesion must be visually observable and located outside the gastro-intestinal tract. The latter is due to the current meat inspection circular in place in Denmark, which stipulates that lesions located in the gastro-intestinal tract need to be dealt with at meat inspection but do not require submission for testing. The reason for this approach is the presence of TB-like lesions, which are not caused by bovTB but other bacteria such as *Rhodococcus equi* or *M. avium*, in which case the legislation does not require testing or any risk mitigation apart from local condemnation of the affected lymph nodes and organs. If lesions located in the gastro-intestinal tract were considered as suspects, then traditional and visual-only inspection should give higher sensitivity than that estimated in this study, though costs of testing would be higher due to more samples tested. A compromise may consist of using Food Chain Information from the herd of origin to distinguish between low- and high-risk herds, where samples should be taken in the latter. Such information may e.g., be presence of imported animals or camelides on the farm.

The sensitivity of serological tests, *Se_SERO_,* was easier to value, as it depended less on subjective assessments. Still, different methods are available with each their sensitivity. Cardoso-Toset et al. (33) investigated five different serological tests for detection of bovTB in pigs, used as supplement to the *post mortem* meat inspection. Because of uncertainty of which test to use in Denmark, the lowest and highest estimates were applied in the calculation of cost of error. In scenario C and D, only one pig from each batch delivered for slaughter was assumed to be tested using serology.

Regarding *P_spread_*: For further spread to occur, infected pigs should be moved or other infected animals (of other species) should be moved, from which the disease could be transmitted to pigs. An estimate for the probability of spread was measured using Equation 3 and 4. We assumed that up to 100 pigs could be moved per high-risk period to other herds for breeding. Hereby, the probability of spread was calculated as the probability of positive pigs being moved out of the herd. If the disease was introduced by another animal than an infected pig, for instance an infected guard llama, and this animal was moved between herds, the probability of spread could be higher than estimated.

Usually, the movements of pigs in Denmark are by contract between the producer and the individual purchasers, and mixing of pigs from different herds is considered poor management and therefore not recommended. This implies that although between five and 17 infected pigs were assumed to be moved within a production cycle (when assuming a *WHP* of 5%), these pigs would most likely only go to a few farms. This was confirmed by considering the data describing the outbreak of avian TB occurring in Danish pigs in the winter of 2016–17; 74% of the sow herds involved only moved pigs to one herd (33). Data from this outbreak were used to estimate the number of holdings pigs were moved to, *N_received_*, if moved.

Regarding *C_eradication_*: In Denmark, suspicion of bovTB requires reporting to the authority. If the case is confirmed, the authority must decide how the case is handled. Possible solutions are tuberculin test of pigs in the infected herd and culling of infected or multiple animals. Moreover, a trace-back and forth will be undertaken (45). From the interview study of international experts, it was seen that Belgium had a more widespread eradication program compared to the contingency program in Denmark. The experience from Belgium is that all cattle on the infected cattle farm and on its contact farms were tested once the outbreak was detected and additionally three times during a 5 year follow-up period. Only cattle farms were included in the Belgian eradication program, as bovTB is not considered an issue in pigs. In Austria, eradication also involves more farms than the Danish legislation suggest. A difference between Denmark and Austria is that in Austria it is common to pasture cattle in certain Alp valleys, where bovTB is known for being present in the wildlife. Cattle kept in these areas are tested independent of lesions of bovTB. Both Belgium and Austria have kept their OTF status, despite the positive cases, due to a low prevalence. United Kingdom is not OTF; 3,578 UK cattle herds were not officially TB free by the end of 2016, due to a bovTB incident (46). Based on the estimates given in the interviews, United Kingdom spends less per infected farm than Belgium and Austria, but in total the costs to eradication exceeds the other countries by far.

The Danish legislation allows the eradication to be tailored to the specific case (45) and no detailed description of an eradication plan is available, since it was last put into action in 1988 (Personal communication, B.B. Jørgensen, Ministry of Environment and Food of Denmark, 2017). Hence, there is some uncertainty related to the cost estimates for eradication, also because the data we have used are referring to cattle farms during an eradication phase. Still, these were the best estimates we could find. Therefore, the figures should be interpreted with care.

Estimates and assumptions for calculation of the impact on trade *C_trade_* related to cases of bovTB in pigs was based bovTB in cattle. It was not possible to base the estimates on bovTB in pigs on literature, as literature on bovTB in pigs and historical data hereof were scarce. Consequently, the cost of bovTB was calculated under the assumption that the reaction to bovTB in pigs would be similar to the reaction of bovTB in cattle. This assumption may have led to overestimation of the expected costs.

### Perspectives

Meat inspection is undertaken to ensure that the meat delivered to consumers is safe and wholesome. Moreover, meat inspection is used to ensure the health and welfare of the slaughtered animals – including detection of notifiable diseases such as bovTB. To meet these objectives, legislation is put in place with individual variations all over the world reflecting both true and perceived differences in risk. This implies that for countries exporting, meat inspection is also undertaken to obtain acceptance of equivalence of the trade partners.

Denmark has a large export of pig meat. Therefore, acceptance of equivalence from important trade partners is of paramount importance before any change in meat inspection will be implemented. Risk assessments have proven to be an effective instrument to illustrate *pros* and *cons* related to palpation and incisions into the various organs (13, 14). When considering replacing TMI with VOI of finisher pigs from the non-controlled housing compartment, bovTB has been identified to be the main hazard, for which there may be a risk of overlooking cases (13). Others have suggested *Toxoplasma gondii* and *Trichinella spp*. (19). However, the latter two hazards cannot be detected macroscopically at meat inspection anyway. Therefore, the specific type of meat inspection is not relevant. Instead, the detection of the presence of these two hazards requires testing. Alternatively, biosecurity level using the concept of controlled housing can be used as an indicator to divide the population into a high- and low-risk group where e.g., only pigs from the high-risk compartment is tested. The latter approach is accepted for *Trichinella spp.*, as it is known that finisher pigs from controlled holdings have a negligible probability of harbouring *Trichinella spp.* (11, 12). The use of controlled housing could also be suggested for *T. gondii,* as studies have shown a very low prevalence of *T. gondii* in finishing pigs raised in controlled housing holdings (47). Regarding avian TB, the prevailing opinion is that it is not a meat-borne hazard. Still, presence of tubercles in a carcass is considered unwanted and should be dealt with. A private standard is in place in Denmark, prohibiting use of peat in pigs unless approved by the health department of the Danish Specific Pathogen Free (SPF-SuS) system (48). This prohibition has lately been extended to also cover the specific type of peat, which caused the outbreak occurring in Denmark in 2016–17; a type of peat which had previously been allowed due to no historic cases of avian TB.

It could be more cost-effective to direct resources to surveillance in imported animals at risk of harbouring bovTB and coming into contact with Danish livestock than to use TMI in pigs from non-controlled housing as a way of surveying for bovTB. The costs associated with such surveillance are expected to be limited, because only a low number of imports is taking place annually; between 2014 and 2016, an average of 366 pigs, 217 cattle (1) and 11 camelides (based upon information from Traces provided by the Danish Veterinary and Food Administration, 2017) were imported into Denmark annually.

## Conclusions

TMI resulted in lowest cost of error, if one incursion of bovTB was expected every year. However, when assuming one introduction in 10 or 30 years, VOI resulted in lowest average annual costs. Additional use of serological testing for bovTB did not contribute to lowering the average annual costs associated with overlooking bovTB in a pig herd.

Should bovTB enter a pig herd belonging to the non-controlled housing compartment, then infection may die out or go unnoticed for some time. If infection does not die out, it will be detected (sooner with TMI, and later with VOI), because of the high number of pigs subjected to meat inspection. Movement of infected pigs to a few other herds may occur before infection is being detected, in particular if VOI is applied. However, pigs act as dead-end hosts for bovTB, and infection is not assumed to be meat-borne. Finally, Denmark has been free from bovTB for more than 30 years among others due to a very low number of imports of livestock for breeding and no import of pigs for slaughter. Therefore, seen on the long perspective it would be cost-effective to replace the current TMI of finishing pigs from non-controlled housing with VOI, although the latter is associated with a lower ability to detect bovTB. Moreover, it could be more cost-efficient to direct resources to bovTB surveillance in imported, high-risk animals coming into contact with Danish livestock.

## Author contributions

LA, RH and LN contributed to the initial design of the work, the collection of data, the simulations and the interpretation of the results, and the drafting of the manuscript. ME and AF contributed mainly to the modelling framework and interpretation of results. BH contributed mainly to the design of the work and the development of the concept of assessment of Cost of Error. All authors contributed to the manuscript and approved the final version.

## Conflict of interest Statement

The authors RH, LN and LA are affiliated with an institution that gives advice to Danish farmers and the agro-food business. ME, AF and BH certify that they have no affiliations with or involvement in any organization or entity with a financial interest or non-financial interest in the subject matter or materials in this manuscript.

The handling Editor declared a past co-authorship with one of the authors LA.

## References

[1] Ministry of Environment and Food of Denmark. Animal Health in Denmark 2016. (2016) https://www.foedevarestyrelsen.dk/Publikationer/Alle%20publikationer/Animal%20Health%20in%20Denmark%202016.pdf.

[2] Jepsen A Meat inspection (in Danish). The Royal Veterinary and Agricultural University, Frederiksberg, Denmark: DSR Forlag (1957).

[3] Bailey SS Crawshaw TR Smith NH Palgrave CJ *Mycobacterium bovis* infection in domestic pigs in Great Britain. Vet J (2013) 198(2):391–7. 10.1016/j.tvjl.2013.08.035 24095608

[4] APHA. TB in Non-Bovine Species. Animal and Plant Health Protection Agency, Great Britain. (2017) https://www.gov.uk/government/statistical-data-sets/other-tb-statistics.

[5] Corner LA The role of wild animal populations in the epidemiology of tuberculosis in domestic animals: how to assess the risk. Vet Microbiol (2006) 112(2-4):303–12. 10.1016/j.vetmic.2005.11.015 16326039

[6] Serraino A Marchetti G Sanguinetti V Rossi MC Zanoni RG Catozzi L Monitoring of transmission of tuberculosis between wild boars and cattle: genotypical analysis of strains by molecular epidemiology techniques. J Clin Microbiol (1999) 37(9):2766–71. 10449449 10.1128/jcm.37.9.2766-2771.1999 PMC85373

[7] Thoen COT Diseases of Swine Leman AD Shaw BE Mengeling WL D´Allaire S Taylor DJ, 7th ed London, United Kingdom: Wolfe Publishing Ltd (1992).

[8] Barron MC Tompkins DM Ramsey DS Bosson MA The role of multiple wildlife hosts in the persistence and spread of bovine tuberculosis in New Zealand. N Z Vet J (2015) 63 Suppl 1(1):68–76. 10.1080/00480169.2014.968229 25384267 PMC4566902

[9] Corner L Melville L Mccubbin K Small KJ Mccormick BS Wood PR Efficiency of inspection procedures for the detection of tuberculous lesions in cattle. Aust Vet J (1990) 67(11):389–92. 10.1111/j.1751-0813.1990.tb03020.x 2085291

[10] EU Commission. Regulation (EC) no 854/2004 of the European Parliament and of the Council of 29 April 2004. (2004) https://www.cefas.co.uk/media/1549/extract_reg_no_854_2004.pdf

[11] EU Commission. Commission Implementing Regulation (EU) 2015/1375 laying down specific rules on official control for *Trichinella* in meat. (2015) http://eur-lex.europa.eu/legal-content/EN/TXT/PDF/?uri=CELEX:32015R1375&rid=1.

[12] Alban L Petersen JV Ensuring a negligible risk of Trichinella in pig farming from a control perspective. Vet Parasitol (2016) 231:137–44. 10.1016/j.vetpar.2016.07.014 27448746

[13] Alban L Vilstrup C Steenberg B Jensen H.E Aalbæk B Thune-Stephensen Assessment of the risk for humans associated with Supply Chain Meat Inspection – The Danish way. Danish Veterinary and Food Administration /DMA. (2008) file:///C:/Users/LF.LiA/Downloads/modernisation-of-meat-inspection-dk.pdf.

[14] Alban L Steenberg B Petersen JV Jensen S Is palpation of the intestinal lymph nodes a necessary part of meat inspection of finisher pigs? Internal report. Danish Agriculture & Food Council. (2010)http://lf.dk/aktuelt/publikationer/svinekod.

[15] Pacheco G Kruse A.B Petersen J.V Alban L Assessment of risk associated with a change in meat inspection – Is mandatory palpation of the liver and lungs a necessary part of meat inspection of finisher pigs? (2013) Internal report. Danish Agriculture and Food Council. Available from: http://lf.dk/aktuelt/publikationer/svinekod.

[16] EFSA. Scientific Opinion on the public health hazards to be covered by inspection of meat (swine). EFSA J (2011) 9(10):2351. 10.2903/j.efsa.2013.3265 PMC7163758 32313569

[17] EU Commission. Regulation (EC) No 218/2014 amending annexes to Regulation (EC) No 853/2004, (EC), No 854/2004 of the European Parliament and of the Council and (EC) Regulation 2074/2005. (2014) https://www.fsai.ie/uploadedFiles/Reg218_2014.pdf.

[18] EFSA. Modelling the impact of a change in Meat Inspection sensitivity on the surveillance of bovine tuberculosis (bTB) at the country level. Supporting Publications (2013). EN-450 p.

[19] EU Commission. Equivalence of revised EU measures as regards visual post-mortem inspection of pigs. Working paper. Ref.Ares(2017)670128-07/02/2017 (2017).

[20] Foddai A Nielsen LR Krogh K Alban L Assessment of the probability of introduction of *bovine *tuberculosis to Danish cattle farms via imports of live cattle from abroad and immigrant workers. Prev Vet Med (2015) 122(3):306–17. 10.1016/j.prevetmed.2015.08.007 26409756

[21] OIE. Disease information – Bovine Tuberculosis. (2016) http://www.oie.int/wahis_2/public/wahid.php/Countryinformation/Animalsituation.

[22] Jordt AM Lange M Kramer-Schadt S Nielsen LH Nielsen SS Thulke HH Spatio-temporal modeling of the invasive potential of wild boar--a conflict-prone species-using multi-source citizen science data. Prev Vet Med (2016) 124:34–44. 10.1016/j.prevetmed.2015.12.017 26775815

[23] Alban L Andersen MM Asferg T Boklund A Fernandez N Goldbach S WildRisk: Classical swine fever and wild boar in Denmark: A risk analysis. Danish Institute for Food and Veterinary Research (2005). http://orbit.dtu.dk/files/3390946/WILDRISK_2005.pdf.

[24] Jenkins D Guard Animals for Livestock Protection - Existing and Potential Use in Australia. NSW Agriculture 2003. (2003) https://www.dpi.nsw.gov.au/__data/assets/pdf_file/0006/178908/guard-animals.pdf.

[25] Cameron AR The consequences of risk-based surveillance: developing output-based standards for surveillance to demonstrate freedom from disease. Prev Vet Med (2012) 105(4):280–6. 10.1016/j.prevetmed.2012.01.009 22305852

[26] Oorburg D Slaughterhouse information to help improve farm’s health status. Workshop held in connection with 12th Safepork Conference in Brazil (2017) http://www.safepork2017.com.br/programme.php?cat=1.

[27] Martin PA Cameron AR Greiner M Demonstrating freedom from disease using multiple complex data sources 1: a new methodology based on scenario trees. Prev Vet Med (2007) 79(2-4):71–97. 10.1016/j.prevetmed.2006.09.008 17224193

[28] Calvo-Artavia F Alban L Nielsen L Evaluation of surveillance for documentation of freedom from bovine tuberculosis. Agriculture (2013) 3(3):310–26. 10.3390/agriculture3030310

[29] EU Commission. Council Directive 98/46/EC of 24 June 1998 amending Annexes A, D (Chapter I) and F to Directive 64/432/EEC on health problems affecting intra-Community trade in bovine animals and swine. Luxembourg: EU Publications (1998).

[30] EFSA. External Scientific Report of the Contribution of Meat Inspection to Animal Health Surveillance in Bovine Animals. Parma, Italy: Supporting Publications (2012). EN-322.

[31] Sergeant E Happold J Hutchison J Langstaff I Evaluation of Australian surveillance for freedom from bovine tuberculosis. Report Prepared for the Australian Biosecurity CRC for Emerging Infectious Disease. Aust Vet J (2010):42 pp. 10.1111/avj.12648 29243239

[32] Cardoso-Toset F Luque I Carrasco L Jurado-Martos F Risalde MÁ Venteo Á Evaluation of five serologic assays for bovine tuberculosis surveillance in domestic free-range pigs from southern Spain. Prev Vet Med (2017) 137(Pt A):101–4. 10.1016/j.prevetmed.2016.12.016 28089289

[33] Hansen RK Alban L Nielsen LH Dahl J Public supervision due to tuberculosis-like changes in pigs after use of untreated peat (In Danish). (2017). 9 pp p.

[34] Boklund A Toft N Alban L Uttenthal Å Comparing the epidemiological and economic effects of control strategies against classical swine fever in Denmark. Prev Vet Med (2009) 90(3-4):180–93. 10.1016/j.prevetmed.2009.04.008 19439381

[35] Danish Agriculture and Food Council. Statistics 2016 – Pigmeat June 2017. (2017) http://www.lf.dk/tal-og-analyser/statistik/svin/statistik-svin/statistik-svin-2016.

[36] Ministry of Environment and Food of Denmark. Meat Inspection Circular No. 9611 of December 16, 2011. (2011) https://www.retsinformation.dk/Forms/R0710.aspx?id=139770.

[37] Macdiarmid SC Future options for brucellosis surveillance in New Zealand beef herds. N Z Vet J (1988) 36(1):39–42. 10.1080/00480169.1988.35472 16031432

[38] Cameron A Njeumi F Chibeu D Martin T Risk-based disease surveillance - A manual for veterinarians on the design and analysis of surveillance for demonstration of freedom from disease. Rome, Italy: Food and Agriculture Orgaization of the United Nations (2014). 215 p. http://www.fao.org/3/a-i4205e.pdf.

[39] Thurmond MC Conceptual foundations for infectious disease surveillance. J Vet Diagn Invest (2003) 15(6):501–14. 10.1177/104063870301500601 14667012

[40] Foddai A Stockmarr A Boklund A Evaluation of temporal surveillance system sensitivity and freedom from bovine viral diarrhea in Danish dairy herds using scenario tree modelling. BMC Vet Res (2016) 12(1):118 10.1186/s12917-016-0744-2 27323903 PMC4915143

[41] Ellebroek L Mateus A Stärk K Alonso S Lindberg A Contribution of meat inspection to animal health surveillance in Swine. External scientific report submitted to EFSA (2011):80 pp.

[42] Morris RS Pfeiffer DU Jackson R The epidemiology of *Mycobacterium bovis* infections. Vet Microbiol (1994) 40(1-2):153–77. 10.1016/0378-1135(94)90053-1 8073623

[43] Welby S Govaerts M Vanholme L Hooyberghs J Mennens K Maes L Bovine tuberculosis surveillance alternatives in Belgium. Prev Vet Med (2012) 106(2):152–61. 10.1016/j.prevetmed.2012.02.010 22398252

[44] Hill A Brouwer A Donaldson N Lambton S Buncic S Griffiths I A risk and benefit assessment for visual-only meat inspection of indoor and outdoor pigs in the United Kingdom. Food Control (2013) 30(1):255–64. 10.1016/j.foodcont.2012.04.031

[45] Rivière J Le Strat Y Dufour B Hendrikx P Sensitivity of bovine tuberculosis surveillance in wildlife in France: a scenario tree approach. PLoS ONE (2015) 10(10):e0141884 10.1371/journal.pone.0141884 26517372 PMC4627846

[46] Ministry of Environment and Food of Denmark. Decree No. 1417 of December 11, 2007 on bovine tuberculosis in animals susceptible to infection with Mycobacterium Bovis. Denmark: Ministry of Environment and Food of Denmark (2007).

[47] APHA. Statistical data set - Tuberculosis (TB) in cattle in Great Britain. Washington, DC. United States: APHA (2017). https://www.gov.uk/government/statistical-data-sets/tuberculosis-tb-in-cattle-in-great-britain.

[48] Kofoed KG Vorslund-Kiær M Nielsen HV Alban L Johansen MV Sero-prevalence of Toxoplasma gondii in Danish pigs. Vet Parasitol (2017) 10:136–8. 10.1016/j.vprsr.2017.10.004 31014586

[49] SEGES - Danish Pig Research Center. Danish Product Standard. Denmark: SEGES (2017). http://www.pigresearchcentre.dk/~/media/Files/DANISH/DANISH%20produktstandard/Produkt_Standard_UK.pdf.

